# Understanding the Role of Endothelial Cells in Glioblastoma: Mechanisms and Novel Treatments

**DOI:** 10.3390/ijms25116118

**Published:** 2024-06-01

**Authors:** Gabrielle Hovis, Neha Chandra, Nidhi Kejriwal, Kaleb Jia-Yi Hsieh, Alison Chu, Isaac Yang, Madhuri Wadehra

**Affiliations:** 1 Department of Neurosurgery, David Geffen School of Medicine, University of California-Los Angeles, Los Angeles, CA 90095, USA; 2 Department of Pathology and Laboratory Medicine, David Geffen School of Medicine, University of California-Los Angeles, Los Angeles, CA 90095, USAhsiehkj@uci.edu (K.J.-Y.H.); 3 Division of Neonatology and Developmental Biology, Department of Pediatrics, David Geffen School of Medicine, University of California-Los Angeles, Los Angeles, CA 90095, USA; 4 Department of Radiation Oncology, David Geffen School of Medicine, University of California-Los Angeles, Los Angeles, CA 90095, USA; 5 Department of Head and Neck Surgery, David Geffen School of Medicine, University of California-Los Angeles, Los Angeles, CA 90095, USA; 6 Lundquist Institute, Harbor-UCLA Medical Center, Torrance, CA 90502, USA; 7 Jonsson Comprehensive Cancer Center, University of California-Los Angeles, Los Angeles, CA 90095, USA

**Keywords:** glioblastoma, endothelium, angiogenesis, tumor marker

## Abstract

Glioblastoma is a highly aggressive neoplasm and the most common primary malignant brain tumor. Endothelial tissue plays a critical role in glioblastoma growth and progression, facilitating angiogenesis, cellular communication, and tumorigenesis. In this review, we present an up-to-date and comprehensive summary of the role of endothelial cells in glioblastomas, along with an overview of recent developments in glioblastoma therapies and tumor endothelial marker identification.

## 1. Introduction

Glioblastoma is a highly aggressive neoplasm that accounts for 14.5% of all central nervous system (CNS) tumors and 48.6% of CNS malignancies [1]. Within the complex tumor microenvironment (TME) of glioblastomas, endothelial tissue contributes to angiogenesis, cellular communication, and tumorigenesis. Several anti-angiogenic and other endothelial targets have been identified in glioblastomas. In this review, we aim to provide a comprehensive summary of the role of endothelial cells (ECs) in glioblastomas and provide the most recent and significant developments in tumor endothelial marker identification.

## 2. Cells of the Central Nervous System

### 2.1. Endothelial Cells

**Figure 1:**
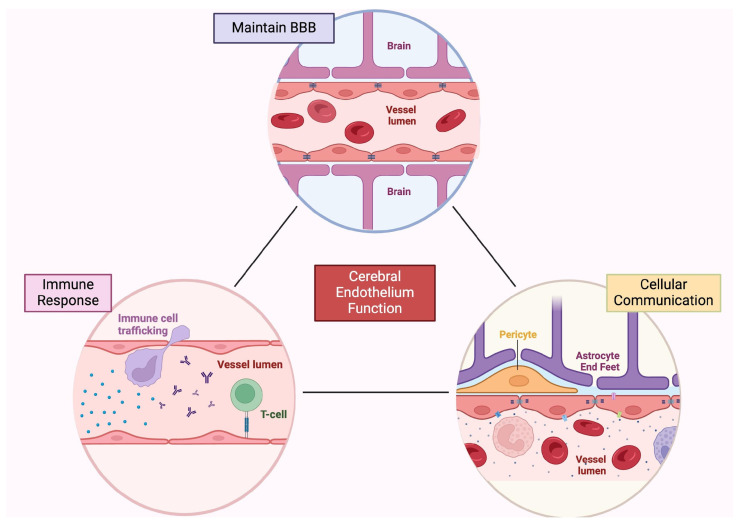
Cerebral endothelial tissue functions. In the brain, the endothelium has many roles, including neuroprotection, inflammatory response, and cellular communication. Endothelial cells help form and maintain the blood–brain barrier, and modulate immune cell trafficking, antigen presentation, and response to inflammatory factors. In addition, endothelial cells participate in intercellular communication through extracellular vesicles, soluble mediators, and growth factors. Created with BioRender.com. BBB, blood–brain barrier.

Blood vessels deliver nutrients to tissues throughout the body and primarily consist of two main cell types: ECs, which form the walls of the vessels and come into contact with the blood, and mural cells (e.g., vascular smooth muscle cells and pericytes), which rest on the outside of the luminal surface. This vascular organization, unique to the brain, is commonly referred to as the blood–brain barrier (BBB), and it is primarily manifested by ECs. However, the organization is maintained by critical interactions with mural cells as well as astrocytes to form the neurovascular unit (Figure 1) [2,3,4,5]. The microvasculature within the CNS is continuous and non-fenestrated, meaning that the vessels have a complete basement membrane (BM) and lack fenestra (pores) in their plasma membrane. This structure enables unique properties to restrict barrier capacity and regulate the passage of ions, molecules, and cells between the blood and the brain [6].

ECs are mesodermally derived, modified simple squamous epithelial cells, and the ECs of the CNS differ both morphologically and functionally from ECs found in the periphery. CNS ECs are sealed together by junctional complexes (tight, adherens, and gap junctions) between the ECs, creating distinct luminal and abluminal membrane compartments. The seals created by tight junctions restrict the paracellular flux of solutes by CNS ECs [7]. In addition, the lack of fenestra on CNS ECs limits the exchange of molecules between the brain tissue and blood. These morphological differences restrict transcytosis in CNS ECs, and extremely low rates of transcytosis are observed in the CNS relative to the periphery [8]. Finally, CNS ECs express low levels of leukocyte adhesion molecules, limiting the entry of immune cells into the brain parenchyma [9].

### 2.2. Neurons

The relationship between the neuronal parenchyma and endothelial tissue is vital to cerebral function and homeostasis [10]. In a process termed neuronal coupling, ECs modulate the diversion of cerebral blood flow (CBF) to areas of neuronal activation [10,11] through the production and release of vasoactive signals such as nitric oxide [11,12]. The induction of angiogenesis by ECs promotes neurogenesis, as this nutrient supply is critical to neuron growth and proliferation [10,13]. The intimate relationship between angiogenesis and neurogenesis is particularly prominent following ischemic stroke [10,13] and within the TME [14]. Additionally, cerebral ECs have a neuroprotective function through the secretion of brain-derived neurotropic factor (BDNF) [10]. BDNF is a neurotrophin which promotes the survival and differentiation of neuroprogenitor cells and modulates neuroplasticity and synaptic transmission [15]. Through the regulation of CBF, pro-angiogenic signal production, and the release of BDNF, cerebral ECs play a central role in neural growth, proliferation, function, and survival.

### 2.3. Mural Cells

Mural cells, which include smooth muscle cells and pericytes, are located on the abluminal surface of ECs. Mural cells regulate several functions within the brain, including the control of resting cerebral blood flow, neurovascular coupling, BBB development and maintenance, and neuronal survival [16,17,18,19,20]. Vascular smooth muscle cells can be found within larger arteries and veins. These cells remain physically separated from the endothelium by an intimal layer of extracellular matrix (ECM). In contrast, pericytes are vascular mural cells embedded in the same basement membrane as ECs and thus constitute a major component of the BBB. In capillaries within the CNS, pericytes are found between ECs and astrocytes, where they occur at a 1:1 ratio compared to ECs [21]. This differs from sites outside the CNS where the ratio of ECs outnumbers pericytes (e.g., 10:1 ratio in the lung or skin) [22]. To date, pericyte research remains challenging due to the lack of clear discrimination criteria versus other mural cells, but some markers that have been used to identify them include platelet-derived growth factor receptor β (PDGFRβ), neural glial antigen 2 (NG2), CD146, and aminopeptidase N [23,24].

### 2.4. Astrocytes

The final components of the neurovascular unit are astrocytes. Astrocytes provide an essential role in supporting and maintaining the BBB [25], and together with the ECs and mural cells, they play an important role in modulating the rate of blood flow to the brain [26]. This occurs, in part, by the extension of endfeet or large, flattened processes that wrap around the blood vessel, and by and large, all gray matter astrocytes are connected to at least one blood vessel [27]. Astrocytes also secrete basement membrane proteins, which together with the astrocytic endfeet ultimately cover up to ~99% of the cerebrovascular surface [21,28].

Functionally, astrocytes secrete a number of critical paracrine factors that act on ECs to change barrier properties. For example, astrocytes secrete Sonic hedgehog, which acts on ECs to promote and maintain BBB formation and integrity throughout development [29]. Additionally, it has been shown that astrocytes can produce angiopoetin-1 which can alter junctional protein expression and endothelial permeability [30]. In this way, astrocytes act as a link between the vasculature and neurons by helping to regulate the passage of metabolites necessary to maintain CNS homeostasis. This link becomes dysregulated in injury, ultimately leading to BBB remodeling. This can either be protective or induce EC apoptosis and decrease the expression of proteins involved in BBB integrity and permeability [26].

### 2.5. Malignant Transformation

Glioblastoma is a highly aggressive malignancy with a median overall survival rate of 15 months and a 5-year, real-world survival rate of 6.8% [31]. Over the past decade, a significant amount of research has been conducted to uncover genetic mutations and identify novel therapeutic avenues. In light of this, the updated 2021 WHO recommendations combined molecular and histologic features to diagnose glioblastoma based on isocitrate dehydrogenase (IDH) status, with glioblastoma diagnosed as IDH wildtype and showing either necrosis, microvascular proliferation, TERT promoter mutation, or EGFR gene amplification [32]. In addition, the methylation of the O6-methylguanine-DNA methyltransferase (MGMT) promoter, an enzyme that is responsible for DNA repair, is assessed to determine the efficacy of alkylating agent chemotherapy [33].

To date, glioblastomas remain relatively resistant to current therapies, including concurrent radiotherapy and the alkylating agent temozolomide (TMZ). The poor therapeutic response may be explained by the high degree of intratumoral heterogeneity, the leaky and tortuous blood vessels, and the existing BBB that surrounds invasive cells [22]. Additionally, glioblastoma cells are capable of a rapid invasion of both local and distant tissues, contributing to treatment resistance and high recurrence rates [34]. While specific migration patterns vary between glioblastoma cell lines, these tumor cells typically exhibit unique perivascular migration, along with the infiltration of white matter tracts [34,35]. To date, there does not appear to be one cell type of malignant glioma origin. Gliomas may arise from adult neural stem cells or multipotent neural progenitor cells that persist within the human CNS [36], but they have also been shown to arise from more differentiated lineages within the brain, including select oligodendrocyte precursor cells, astrocytes, and even mature neurons [37,38]. This adds to the complexity of the disease as well as how these cells interact with the TME.

### 2.6. Tumor Vasculature

Glioblastoma is highly vascular, which distinguishes it from more benign brain tumors, such as low-grade gliomas and meningiomas [39]. The brain tumor vasculature contains two distinct types of vessels: (1) new vessels formed by angiogenesis (neoangiogenic vessels), and (2) pre-existing vessels that may be co-opted by tumor cells (co-opted vessels). For vessels that form via neoangiogenesis, a high degree of vascularization or hypervascularization occurs as a consequence of rapid tumor growth. In this type of vascularization, vessel growth occurs by the proliferation and migration of ECs from preexisting vessels, causing them to sprout. This hypervascularization directly feeds tumor cells by providing oxygen and nutrients, and it also promotes tumor cell progression and invasion. In contrast, during vascular co-option, cancer cells migrate along pre-existing blood vessels to grow and invade the surrounding tissue [40]. This mechanism has been described in brain tumors from histopathological specimens, and it is believed that the cancer cells compress the co-opted vessel, ultimately creating a hypoxic tumor core. The hypoxic tumor environment and resultant glucose deprivation in turn then promote neovascularization through ischemia- or stress-induced angiogenesis [41]. Vascular endothelial growth factor (VEGF) expression is regulated in response to cytokine, growth factor, and hormonal signaling, and VEGF in turn acts on ECs to control angiogenesis. In the case of hypoxia, the hypoxia-inducible factor 1 (HIF-1) accumulates and promotes the transcription of VEGF [41].

In addition, several studies report the involvement of vasculature in supporting tumor signaling pathways. The tumor vasculature helps to assemble the microenvironment by bridging tumor cells with immune cells that have infiltrated the space as well as connecting glioblastoma stem cells (GSCs) and the ECM to ultimately drive tumor progression [42]. Several changes have been described to both the vasculature and the tumor, whereby each begins to resemble the other. Regarding the vasculature, the endothelium undergoes many modifications in the setting of an intracranial tumor. One such change is termed the “endothelial-to-mesenchymal transition” (EndoMT), which involves cellular transition into a mesenchymal-like cell and has been characterized by EC invasion into underlying tissue as enabled by disrupted intercellular connections and the loss of endothelium-specific markers [43]. The cells that have undergone EndoMT show a decreased expression of genes specific to ECs (e.g., CD31) and an increased expression of mesenchymal-specific genes such as fibroblast-specific protein 1 and α-smooth muscle actin [44]. In addition, the mesenchymal-epithelial transition factor (c-MET)/ETS1/MMP-14 axis is specifically associated with EndoMT. The inhibition of c-MET, such as through TMZ administration, reduces vascular formation and tumor growth [43].

In contrast to EndoMT, vascular mimicry refers to the ability of cancer cells to organize into vessel-like structures as an independent means to obtain nutrients and oxygen. The arrangement of these modified vessels mimics features of embryonic vasculogenesis, suggesting that some malignant glioblastoma cells acquire an embryonic-like phenotype [45]. These embryonic-like cells can transform into modified ECs, but they still carry the same genomic alteration as the tumor, suggesting that a portion of the vascular endothelium may be of neoplastic origin [46]. Multiple signaling pathways have been implicated in this process by promoting the increased expression of EC characteristics in tumor cells, including VE-cadherin and VEGFR2 signaling, and the integrin B8-TGFβ1 axis [46,47]. The transformed cells join and recruit pericytes to create networks resembling vessels. It has been shown that these tumor-derived vessels can disrupt the BBB, lead to a loss of neurons and astrocytic endfeet, and even increase permeability to immune cells [3]. These collective features of malignant gliomas have been proposed to contribute to the failure of anti-angiogenic therapy [48].

### 2.7. Structural Communication between TCs and ECs

Glioblastoma tumor cells and ECs may communicate with one other using structural mechanisms, such as gap junctions and extracellular vesicles (EVs). Gap junctions are intercellular channels composed of integral membrane proteins called connexins [49], which facilitate communication between tumor cells and ECs. The exchange of VEGF and other pro-angiogenic cytokines through gap junctions may promote tumorigenesis [50,51].

Another way that glioblastoma tumor cells and ECs communicate is through the exchange of EVs, membrane-bound vesicles secreted from cells that aid in bidirectional, intercellular communication [52]. EVs have been shown to reflect and influence the phenotype of the cells within the TME. By delivering a diverse collection of genomic, lipidomic, and proteomic material to nearby and distant cells, EVs can alter the phenotype and function of the recipient cell. As such, EVs have been shown to promote angiogenesis, suppress the immune system, alter tumor cell invasion and migration, and confer drug resistance, promoting glioblastoma recurrence [53]. Specifically, EVs released by glioblastoma tumor cells can carry VEGF, transforming growth factor-β (TGF-β), chemokines, and proteolytic enzymes as cargo, all of which are pro-angiogenic factors that play an active role in altering tumor vasculature [54,55]. Donor cells (tumor cells or surrounding cells in the stroma) release EVs, which then fuse with ECs and alter transcriptomic expression. EC-induced angiogenesis then begins at the site of fusion through the activation of the transforming AKT/β-catenin pathway [56,57].

## 3. Angiogenesis in Glioblastoma

### 3.1. Angiogenesis Mechanism

**Figure 2:**
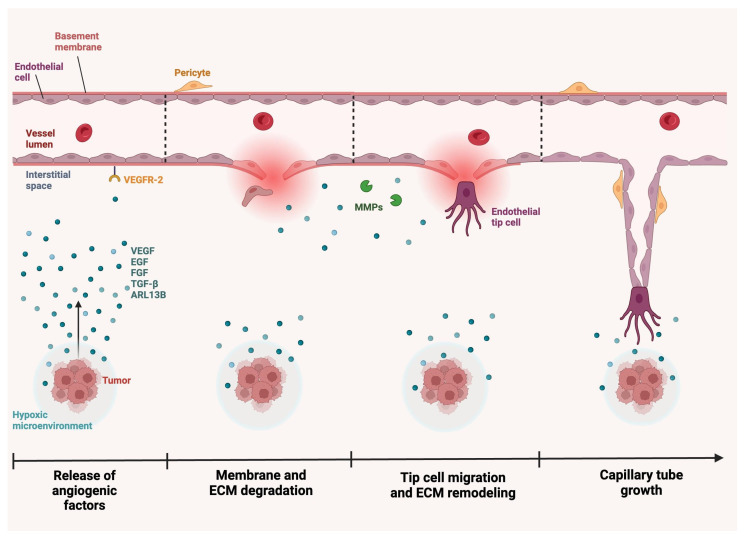
Tumor neovascularization in glioblastomas. In the setting of a tumor, stresses such as hypoxia prompt the release of signaling proteins, such as VEGF, ARL13B, TGF-β, FGF, and EGF. These proteins interact with receptors, such as VEGFR-2, on the endothelial cell membrane. This interaction prompts several downstream effects, including degradation of the basement membrane and ECM, fibroblast displacement, and endothelial cell invasion of the stroma. Over time, MMPs and other proteins remodel the ECM, and endothelial tip cells migrate to the end of budding vessels. Guided by VEGF gradients, these tip cells aid in the formation of a new basement membrane and capillary with the incorporation of endothelial cells, smooth muscle cells, and pericytes. Created with BioRender.com. ARL13B, ADP-ribosylation factor-like GTPase 13B; ECM, extracellular matrix; EGF, epidermal growth factor; FGF, fibroblast growth factor; MMP, matrix metallopeptidase; TGF-β, transforming growth factor-β; VEGF, vascular endothelial growth factor; VEGFR-2, vascular endothelial growth factor receptor-2.

Angiogenesis describes the growth and differentiation of vascular ECs, which is mediated by various biochemical, mechanical, and chemotactic signals [58,59,60,61]. As neovascularization is essential to tumorigenesis, angiogenesis is closely associated with neoplastic growth, progression, and invasion [62,63,64]. During angiogenesis, ECs are activated and show increased permeability and proliferation. In response to oncogenic signals such as VEGF or TGF-β, matrix metalloproteinases (MMPs) induce the degradation and remodeling of the ECM and the endothelial cell basement membrane, after which ECs invade the stroma. Finally, a basement membrane is formed around the ECs, smooth muscle cells, and pericytes to create a new capillary (Figure 2) [65,66]. In glioblastomas, multiple angiogenic factors act on ECs to spur this process.

**Figure 3:**
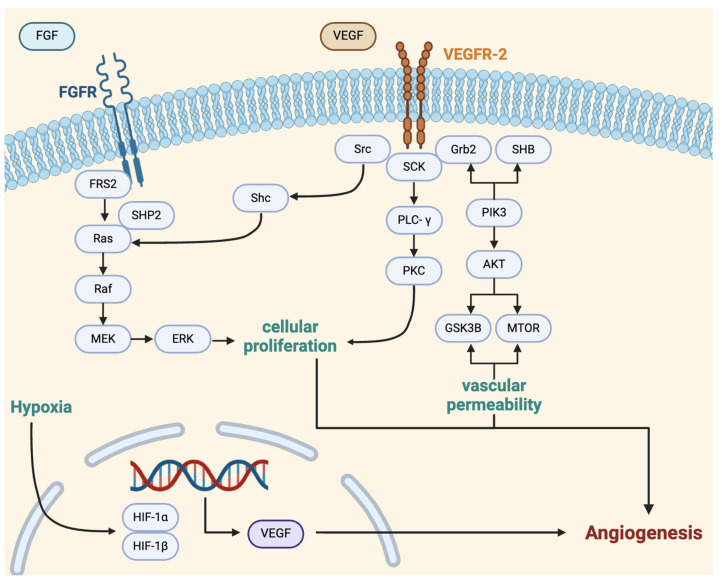
VEGF signaling pathway. VEGF binds to VEGFR-2 receptors on the surface of endothelial cells to promote cellular proliferation and migration, vascular permeability, and gene expression. Ultimately, VEGF induces angiogenesis through numerous pathways. This figure provides a simplification of VEGF signaling in angiogenesis with mention of key factors and pathways, including Src, SCK, Grb2, SHB, Ras-Raf-MEK-ERK, PLCγ/PKC, and PI3K-Akt [58,72]. Created with BioRender.com. VEGF, vascular endothelial growth factor; VEGFR, vascular endothelial growth factor receptor.

VEGFs are major drivers of angiogenesis, and the family is composed of several well-studied pro-angiogenic signaling molecules [67,68,69,70,71], which play critical roles in tumor invasion and progression. VEGF-A and VEGF-B are the primary pro-angiogenic factors of this family. These ligands can exert various effects and operate primarily by binding to vascular endothelial growth factor receptors (VEGFRs), which are found in distinct cell populations. For example, VEGFR-1 is found on blood vascular ECs, while VEGFR-2 is expressed on both growing blood vasculature and lymphatic vessels [69]. The activation of the VEGFR promotes cellular proliferation, vascular permeability, and gene expression through interaction with signaling proteins, including Src, SCK, Grb2, and SHB, and pathways, such as Ras-Raf-MEK-ERK, PLCγ/PKC, and PI3K-Akt (Figure 3) [72]. These pathways also play other roles in glioblastoma tumorigenesis, such as the dysregulation of the Ras-Raf-MEK-ERK pathway, which may result in the ability of tumor cells to bypass the autophagy process and exhibit greater cellular proliferation and differentiation [73,74].

Multiple pro-angiogenic factors that target ECs have been identified in glioblastomas (Table 1). Among the best-studied factors is TGF-β, which is known to promote angiogenesis via the activation of its type II receptor, TβRII. TGF-β can be produced by tumor cells, as well as surrounding cells in the TME, such as leukocytes and stromal cells. A recent gene expression profiling study found that TGF-β is involved in multiple stages of tumorigenesis, from blood vessel development to ECM organization (Figure 2) [75]. TGF-β acts on fibroblasts and epithelial cells to induce VEGF production, and TGF-β-induced VEGF binds VEGFRs on ECs, resulting in EC proliferation and migration [76]. Additionally, TGF-β can modify the ECM by inducing MMP activity, which promotes EC migration, an essential process for angiogenesis (Figure 2). Overall, this factor activates glioblastoma tumor cells to increase VEGF-A production, which can then bind to ECs to promote capillary formation [77]. TGF-β-induced Smad2 phosphorylation levels are high in the vascular compartment of tumor tissue and its expression is correlated with increased tumor volume and reduced survival in mice [78].

Another prominent growth factor that has been shown to increase VEGF-A levels is fibroblast growth factor-2 (FGF2). Tumor-secreted FGF2 promotes angiogenesis by stimulating ECs to produce VEGF [79,80]. The activation of FGF2 also promotes EC proliferation through the Ras-Raf-MEK-ERK signaling pathway (Figure 3) [81]. The inhibition of FGF2 signaling has been associated with the suppression of glioblastoma cell proliferation [82] and reduced angiogenesis in glioblastomas [83].

The four major VEGF proteins bind to distinct VEGF receptors (VEGFRs). Within tumorigenesis, VEGF-A has been best characterized, and it is considered to be a strong prognostic marker for glioblastoma aggressiveness, given that patients with the highest circulating levels of VEGF-A have the worst overall survival [84,85]. Secreted by both tumor cells and stromal cells (fibroblasts, macrophages, and EC), VEGF-A binds to VEGFR-1 and VEGFR-2 on tumor ECs, promoting their proliferation and stimulating angiogenesis. VEGF-A stimulates EC migration and activates multiple pathways to promote EC proliferation, survival, nitric oxide production, and angiogenic response [86,87,88]. Endothelial tip cells, the leading cells at the tips of budding blood vessels, highly express VEGFR-2; this allows them to guide the growth of new vasculature along the VEGF-A gradient generated by tumor cells. This migration is facilitated by dynamic filopodia located at the growing end of endothelial tip cells [89,90]. In contrast, VEGF-B has a more indirect pro-angiogenic function by improving vascular survival [91,92]. At high FGF2/FGFR1 expression levels, VEGF-B may have an anti-angiogenic effect through the inhibition of FGF and FGFR, but this mechanism is poorly understood [92].

In addition to the VEGF family, multiple other pro-angiogenic factors influence VEGFR signaling directly or indirectly. One well-characterized protein is ADP-ribosylation factor-like GTPase 13B (ARL13B). An increased EC expression of ARL13B has been correlated with a poor prognosis in glioblastomas [93]. This GTPase is typically involved in cilia production and structure, and it is also highly expressed in glioblastoma cells [94]. ARL13B also interacts with the intracellular domain of VEGFR2 on ECs to promote VEGFA-VEGFR2 signaling [93]. By increasing the activity of the VEGFA-VEGFR2 pathway, ARL13B stimulates angiogenesis and glioblastoma tumor growth.

Epidermal growth factor (EGF) is highly expressed in tumor, rather than normal, ECs. EGF is released by tumor cells and binds to the EGF receptor (EGFR) on tumor ECs to promote EC migration and capillary tube formation [95]. EGF operates independently of VEGF but induces comparable capillary formation through alternate pathways involving PI3K, MAP kinase (MAPK), and eNOS [88,96]. In normal ECs, the oncogene ErbB2 is activated by EGF. In tumor ECs, increased EGFR and reduced ErbB3 expression result in greater cellular proliferation and vascular growth [95]. As angiogenesis plays a critical role in tumor growth, the search for anti-angiogenic targets is prominent in glioblastoma research.

### 3.2. Anti-Angiogenic Therapies in Glioblastoma

Given the key role of angiogenesis in tumor growth and metastasis [62,63,64], and the highly vascular nature of glioblastomas [97], anti-angiogenic drugs, particularly those that target VEGF, have been extensively explored as management options [90]. We identified 56 clinical trials reported in the ClinicalTrials.gov database using the search terms “glioblastoma multiforme” and “endothelial cells” and a start date of 2000 or later. Of these studies, 29 clinical trials were relevant to our subject of interest (Table 2).

Common anti-VEGF therapies include Minocycline, Sorafenib, and Bevacizumab [98]. Minocycline has been found to increase HIF-1α protein degradation through increased coupling between HIF-1α and the von Hippel–Lindau protein, in turn suppressing EC neovascularization independent of the Akt/mTOR pathway [99]. Minocycline is typically administered in a combination of nine repurposed drugs that inhibit glioblastoma cell growth and signaling pathways known as Comprehensive Undermining of Survival Paths (CUSP)9v3 [100]. CUSP9v3 reduced the viability of glioblastoma cells and inhibited three-dimensional tumor growth, leading to enhanced apoptosis. The treatment regimen showed a survival benefit, but only nine patients were eligible for analysis, and it is unclear whether the enhanced therapeutic effect is primarily due to a single drug or the combination of drugs in the CUSP9v3 regimen [101,102].

In contrast to Minocycline, which decreases HIF-1a expression, Sorafenib is a tyrosine kinase inhibitor that blocks VEGFRs and is thought to inhibit cell growth through the suppression of the PI3K/Akt and MAPK pathways [103,104,105]. Sorafenib was thought to initiate apoptosis, but it did not improve sensitivity to radiotherapy or chemotherapy in vivo [106]. Moreover, clinical trials with Sorafenib as a monotherapy and combination therapy have failed [107,108]. This may be due to its poor bioavailability and toxicity [108]. Interestingly, low-density lipoprotein-specific micelles loaded with Sorafenib were found to improve BBB penetration and glioblastoma cell uptake relative to free Sorafenib controls [109]. A phase II study is currently investigating the safety, tolerability, and effectiveness of the combination of three drugs, Sorafenib (Nexavar^®^), Valproic acid (Depakote^®^), and Sildenafil (Viagra^®^) when used to treat glioblastomas (NCT trial number: NCT01817751).

Bevacizumab (BEV) is a monoclonal antibody against VEGF-A, which was approved by the Federal Drug Association in 2009 for the management of recurrent glioblastomas [110,111]. While many studies have demonstrated delayed tumor progression through the inhibition of the VEGF-A and HIF-1a pathway, randomized, placebo-controlled, phase III trials of BEV have not shown an overall survival benefit [90,112,113]. Additionally, when Sorafenib was combined with BEV, patient outcomes did not improve, and circulating ECs increased with disease progression [114]. Despite this, BEV is the most frequently prescribed anti-angiogenic agent for recurrent glioblastomas, as it has been shown to significantly reduce cerebral edema [110].

The treatment resistance of glioblastomas highlights the utility of employing innovative combination therapies to target glioblastomas. Sorafenib and a derivative of coumarins (Osthole) together led to an increase in induced cell apoptosis, which was more effective than TMZ or BEV alone. The combination of both compounds completely inhibited autophagy and limited angiogenesis [115]. In addition, a Phase II combination therapy study with Sorafenib and Erlotinib for patients with progressive or recurrent glioblastomas did not show any significant increase in overall survival time compared to monotherapies (Table 2) [107]. Tomivosertib has been utilized for non-glioblastoma cancer treatment, but, when combined with TMZ, acts as an inhibitor of tumor angiogenesis by targeting both ECs and the angiogenic TME [116]. Notably, treatment resistance and enhanced migration may develop as tumor cells adapt to stressors induced by antineoplastic agents [117,118,119]. Jahangiri et al. identified a c-Met/β1 integrin complex which enables glioblastoma cell resistance to BEV by promoting tumor cell migration and extravasation and improving tolerance to hypoxic environments [117]. The identification of markers such as the c-Met/β1 integrin complex may serve as targets for new drug development to improve glioblastoma management.

The lack of success for agents targeting VEGF/VEGFR2 points to the fact that other factors may play a more influential role in glioblastoma tumorigenesis. One possible alternative is targeting ECs. Tumor ECs generate a VEGF-A-independent pathway of tumor resistance to antiangiogenic treatment [120]. As ECs are known to produce signals that favor tumor growth, it is possible that these tumor-activated ECs contribute to antiangiogenic therapy resistance. For example, the ETS transcription factor (ETV2) is highly expressed in high-grade human glioma. ETV2 is necessary for the differentiation of glioblastoma neural stem cells into ECs, suggesting its involvement in angiogenesis and potential as a therapeutic target [121]. Another pathway involved with EC expression in glioblastomas is the c-MET pathway. The activation of this receptor tyrosine kinase is associated with EndoMT and a decrease in VEGFR expression, which in turn promotes resistance to anti-VEGF therapies [122,123,124]. Antibodies targeting c-MET have been shown to decrease the growth of glioblastoma cells and may show promising results compared to angiogenic therapies [125].

### 3.3. Other Methods to Target Angiogenesis

Cilengitide is a cyclic RGD-containing peptide which targets integrins αvβ3 and αvβ5. Integrins are transmembrane receptors that promote cellular communication, binding to the ECM, and crosstalk between the stroma and tumor cells. They play an important role in cell migration, invasion, and neoangiogenesis. The integrins αvβ3 and αvβ5 are highly expressed in glioblastoma tumor cells, and they have been used as biomarkers to detect glioblastoma tumor cells [126,127]. Data from the phase 2 trials suggested promising antitumor activity as a single agent in recurrent glioblastomas and in combination with TMZ chemoradiotherapy in newly diagnosed, methylated MGMT promoter glioblastomas. However, the addition of Cilengitide to TMZ chemoradiotherapy in the CENTRIC EORTC trial provided no improvement in patient overall survival outcomes [128].

In addition to integrins, some bone morphogenic proteins (BMPs) have been shown to play an active role in glioblastoma pathogenesis, acting on endothelial cells to induce their migration and proliferation. Several different cell types in tumor tissue may secrete BMP, including stromal cells and glioblastoma tumor cells [129]. BMPs are cytokines and members of the Transforming Growth Factor (TGFβ) family [129]. This discussion will focus on BMP 2, 4, and 9, which primarily interact with type I receptors BMPR1a (Alk3) or BMPR1b (Alk6) and BMPRII on ECs [130].

In addition to playing a key role in embryonic development and cell differentiation, recent evidence suggests BMPs may also play a role in cancer progression [131,132]. Some state that BMPs induce tumor cell differentiation, thereby suppressing tumorigenic potential [133]. For instance, BMP 4 halts the cell cycle of glioma cells, thereby decreasing their proliferation [134]. While some data indicate that BMPs have a tumor-suppressing role, much data points to BMPs promoting glioblastoma and invasion. Many BMPs stimulate the VEGF promoter in ECs to promote angiogenesis [135,136]. BMP 2 and 9 have been shown to have an active role in glioblastoma pathogenesis. BMP 2 inhibitors have been shown to decrease tumor growth both in vitro and in vivo in glioblastoma [132]. BMPs also increase glioblastoma invasiveness by promoting tumor cell migration [133,137]. BMP 9 triggers Smad 1, 5, 8 phosphorylation and induces cell cycle progression [138]. BMP inhibitors JL5, DMH1, and Ym155 suppress the growth of glioblastomas. JL5 inhibits type 1 and type 2 BMP receptors, DMH1 inhibits only type 1 BMP receptors, and Ym155 induces BMPR2 degradation [139]. These inhibitors decreased glioblastoma self-renewal and increased tumor cell death.

## 4. Alternative Interventions

### 4.1. Immunotherapy for Glioblastoma

While immunotherapeutic agents have transformed treatment for several cancers, the traditional checkpoint inhibitors have thus far shown marginal results in glioblastomas. Randomized phase II trials in patients with recurrent glioblastomas showed ineffective results when an anti-programmed-death 1 (PD-1) blockade was administered as a monotherapy or as a combination therapy with bevacizumab [85]. Nonetheless, finding the right combination of therapies and methods to activate the immune system such as through the use of adoptive cell therapies or therapeutic vaccines continues to hold promise in the search for effective treatments against this aggressive brain tumor [140]. For example, a phase 1 trial evaluating a locoregional delivery of IL-13Rα2-targeting chimeric antigen receptor (CAR)-T cells in recurrent high-grade glioblastomas showed promising clinical activity [141]. Similarly, intrathecal bivalent CAR-T cells targeting EGFR and IL13Rα2 in recurrent glioblastomas reported interim results showing manageable toxicity and encouraging progression-free survival [142]. Despite these early findings, challenges such as tumor heterogeneity, immune evasion mechanisms, and patient selection criteria have hindered the successful translation of these combination approaches into clinically meaningful outcomes [143].

Recent advancements in immunotherapy for glioblastoma have showcased the need to find novel immunological targets for tumors in the brain. GD2 and CD47, for example, are promising targets for immunotherapy. GD2 is a disialoganglioside that is overexpressed in multiple tumor histologies and promotes tumor cell survival. CD47 is a checkpoint inhibitor that reduces macrophage function in tumor cells [144]. Both GD2 and CD47 have been highly expressed in glioblastomas and, when combined, inhibit tumor progression. For example, while anti-GD2 therapy alone lacks significant efficacy, the simultaneous targeting of GD2 and CD47 has shown promise in reducing tumor size [145]. Immune checkpoints such as TIM3, IDO1, LAG3, and CD137 are now becoming novel avenues for potential investigation in glioblastoma immunotherapy. Tumor-associated macrophages (TAMs) contribute significantly to cancer immunosuppression and therapy resistance in glioblastoma, primarily through the secretion of immunosuppressive cytokines. Thus, targeting cytokine IL-6 and stimulating CD40 to expose the glioblastoma to an immune checkpoint blockade, together may mitigate tumor antigen-mediated immune suppression while maintaining T cell infiltration in glioblastoma [145].

A new approach in immunotherapy is the use of a Nano-reshaper to concurrently deliver CBD and LIGHT. The mechanism aims to reprogram both systemic and local immune responses to enhance immunotherapy against glioblastomas. CBD and LIGHT countered systemic and local immunosuppression, enhancing the anti-glioblastoma immune response by elevating systemic T cell counts and facilitating effector T cell infiltration into glioblastomas. The interaction between Nano-reshaper and anti-PD-1 highlights its promising clinical applicability and reinforces the potential of this approach to augment other T cell-based immunotherapies, including vaccines and viruses, against glioblastomas [146].

### 4.2. Nanoparticles and Other Novel Technologies

Treating glioblastomas compared to other solid tumors has unique challenges. Epigenetic remodeling, external stress, and genetic instability contribute to its heterogeneous TME, which in turn poses a challenge in the development of effective therapies [147,148,149]. Moreover, the BBB poses an additional obstacle when designing drug therapies, as it is impermeable to 98% of small-molecule and approximately 100% of large-molecule drugs [150,151]. Given these barriers to treatment, the development of novel treatment modalities and delivery methods may improve the management of glioblastoma.

Advanced therapeutic strategies such as nanoparticles (NPs) have recently been investigated to improve drug efficacy for therapy-resistant glioblastomas. NPs have been shown to enhance drug solubility, reduce off-target toxicity, and promote blood–brain barrier permeability [152,153,154]. Specifically, active rather than passive targeting drug delivery systems supplemented with vectors are used in glioblastoma therapy [155]. Receptor-mediated endocytosis (RME) allows drugs to bind to intracranial ECs and adsorptive-mediated endocytosis (AME), driven by electrostatic interactions, is important for transporting the drugs into the brain, significantly enhancing BBB permeability [156]. Not only have NPs shown improved drug efficacy, but nanotechnology has also revolutionized the detection and screening of brain tumors [157]. NPs demonstrated longer half-times and sustained drug release in tumor sites. For example, a nanoparticle-based carrier was synthesized to increase the stability of TMZ, a chemotherapy subject to rapid hydrolysis and poor solubility in physiological conditions [158]. The promising effects of NPs could be utilized with combination therapies.

## 5. Conclusions

Endothelial cells play a critical role in the tumorigenesis of glioblastoma, including angiogenesis and cellular communication. Multiple therapies have been developed to target ECs in glioblastoma, but to date, anti-angiogenic therapies have not managed to produce durable, long-term responses. There is a need for future research to identify targets and develop effective treatments to optimize the management of glioblastoma.

## Figures and Tables

**Table 1:** Major angiogenic factors targeting endothelial cells in glioblastomas.

Factor	Receptor	Mechanism
VEGF-A	VEGFR-1 and VEGFR-2	Stimulates EC migration, proliferation, survival, nitric oxide production, and angiogenic response
ARL13B	VEGFR-2	Binds the intracellular domain of VEGFR2 on ECs to promote VEGFA-VEGFR2 signaling
TGF-β	TβRII	Acts on both TCs and ECs to induce neovascularization and causes ECM remodeling using MMPs
FGF2	FGFR	Stimulates EC VEGF production and proliferation
EGF	EGFR	Promotes EC migration and capillary tube formation
ETV2	Conserved ETS motifs in the ZRS	Transdifferentiates GBM stem cells to an endothelial lineage
Pleiotrophin	ALK1	Causes VEGF deposition at blood vessels, induces EC proliferation
PDFG-B	PDGF receptor β	Stimulates VEGF production in ECs
Ang-1	EC-specific Tie receptors	Acts on ECs to stimulate angiogenesis and EC survival

VEGF, vascular endothelial growth factor; VEGFR, vascular endothelial growth factor receptor; ARL13B, ADP-ribosylation factor-like protein 13B; TGF-β, transforming growth factor-beta; TβRII, TGF-β type II receptor; FGF2, fibroblast growth factor 2; FGFR, fibroblast growth factor receptor; EGF, epidermal growth factor; EGFR, epidermal growth factor receptor; ETV2, Ets variant 2; ALK1, activin receptor-like kinase 1; PDFG-B, platelet-derived growth factor B; Ang, angiopoietin; ZRS, zone of polarizing activity regulatory sequence.

**Table 2:** Recent glioblastoma clinical trials for endothelial cell-related therapies.

Agent	Target	Combination Therapies	NCT Trial Number	Phase	Status	Outcome (in Experimental Group Relative to Control)	Center or Company Name
Bevacizumab	VEGF	Temozolomide	NCT00590681	Phase II	Completed Sept. 2014	Prolonged PFS, no improvement in OS	University of Chicago, Genentech, Inc., Oceanside, CA, USA
		Sorafenib	NCT00621686	Phase II	Completed Feb. 2014	No improvement in outcome	Alliance for Clinical Trials in Oncology, NCI, Bethesda, MD, USA
		Erlotinib	NCT00671970	Phase II	Completed Apr. 2010	Similar PFS and radiographic response	Duke University, Genentech, Inc., Oceanside, CA, USA
		Rindopepimut (CDX-110)	NCT01498328	Phase II	Completed May 2016	Improved PFS, ORR, and ability to discontinue steroids for ≥6 months	Celldex Therapeutics, Hampton, NJ, USA
		Poly-ICLC	NCT02754362	Phase II	Completed June 2019	Pending	NYU Langone Health, MOUNT SINAI HOSPITAL, New York, NY, USA
		Optune	NCT01925573	Interventional	Completed Aug. 2019	Pending	University of Maryland, Baltimore, NovoCure Ltd., Portsmouth, NH, USA
		Trebananib	NCT01609790	Interventional	Completed May 2022	Shortened PFS	NCI, NRG Oncology, Columbus, OH, USA
		Ascorbic Acid	NCT02833701	Phase I	Completed Mar. 2019	Pending	University of Nebraska, NCI, Bethesda, MD, USA
		Retifanlimab + hypofractionated radiotherapy	NCT06160206	Phase II	Ongoing	Pending	Academic and Community Cancer Research United, NCI, Bethesda, MD, USA
VB-111	Ad-PPE-Fas-c	NA	NCT04406272	Phase II	Ongoing	Pending	Dana-Farber Cancer Institute, VBL Therapeutics, New York, NY, USA
Apatinib	VEGFR-2	Temozolomide	NCT04814329	Observational	Ongoing	Pending	Beijing Sanbo Brain Hospital, Beijing, China
Anlotinib	EGFR	NA	NCT04004975	Phase II	Completed July 2021	Pending	Shandong Cancer Hospital and Institute, Jinan, Shandong, China
		Temozolomide	NCT04547855	Phase II	Ongoing	Pending	The First Affiliated Hospital of Nanchang University, Nanchang, Jiangxi, China
Glasdegib	Sonic hedgehog receptor smoothened (SMO)	Temozolomide	NCT03466450	Phase II	Completed Nov. 2023	Pending	Hospital del Mar, Barcelona, Catalonia, Spain
Temozolomide	DNA (alkylating agent)	Radiation therapy + bevacizumab	NCT00884741	Phase III	Completed Mar. 2013	No improvement in OS	Providence Hospital, Portland, OR, USA
Napabucasin (BBI608)	STAT3	Temozolomide	NCT02315534	Phase II	Completed June 2019	Pending	Laura and Isaac Perlmutter Cancer Center, New York, NY, USA
Thrombospondin-1 analog (ABT 510)	CD36 receptor found on ECs	Radiation	NCT00584883	Phase I	Completed July 2008	Pending	University of Alabama at Birmingham, Birmingham, AL, USA
Cediranib	(VEGFR)-1, VEGFR-2, VEGFR-3	Olapirib	NCT02974621	Phase II	Completed Dec. 2022	Pending	UC San Diego Moores Cancer Center, San Diego, CA, USA
TTAC-0001 (Taniburimab)	VEGFR-2	NA	NCT03033524	Phase II	Completed June 2017	Pending	PharmAbcine, Yuseong-gu, Daejeon, Republic of Korea
		Bevacizumab	NCT03856099	Phase II	Completed July 2022	Pending	Stanford Advanced Medical Center, Palo Alto, CA, USA
		Pembrolizumab	NCT03722342	Phase I	Completed Sept. 2022	Pending	Austin Hospital, Heidelberg, VIC, Australia
Recombinant Human Endostatin	Broad-spectrum angiogenesis inhibitor	Temozolomide + Irinotecan	NCT04267978	Phase II	Ongoing	Pending	Beijing Sanbo Brain Hospital, Beijing, China
EGFR Bi-armed Activated T-cells (BATs)	EGFR	Temozolomide + radiation	NCT03344250	Phase I	Ongoing	Prolonged OS and PFS	University of Virginia, Charlottesville, VA, USA
Nanoparticles	variable	Radiotherapy + Temozolomide	NCT04881032	Phase I/II	Ongoing	Pending	CHU de Brest, Brest, Brittany, France
hrBMP4	VEGF	NA	NCT02869243	Phase I	Completed June 2021	Reduction in tumor growth; 2/15 with complete regression and extended survival	Tel Aviv Sourasky Medical Center, Tel Aviv-Yafo, Israel
Erolotinib	EGFR	Sorafenib	NCT00445588	Phase II	Completed Apr. 2026	No significant survival improvement	University of Alabama at Birmingham, Birmingham, AL, USA

NA, not applicable; NCI, National Cancer Institute; NRG, National Research Group; ORR, overall response rate; OS, overall survival; PFS, progression-free survival; VEGF, vascular endothelial growth factor; Poly-ICLC, polyinosinic-polycytidylic acid-poly-l-lysine carboxymethylcellulose; VB-111, ofranergene obadenovec; Ad-PPE-Fas-c, adenovector that expresses Fas-c under the control of the modified pre-proendothelin-1 (PPE-1) promoter; VEGFR, vascular endothelial growth factor receptor; EGFR, epidermal growth factor receptor; STAT3, signal transducers and activators of transcription 3; hrBMP, human recombinant bone morphogenetic protein.
